# Comparison of transient and permanent LAD ligation in mice using 18F-FDG PET imaging

**DOI:** 10.1007/s12149-022-01734-8

**Published:** 2022-03-30

**Authors:** Maximilian Fischer, Tobias Weinberger, Denise Messerer, Mathias J. Zacherl, Christian Schulz, Steffen Massberg, Peter Bartenstein, Sebastian Lehner, Guido Boening, Andrei Todica

**Affiliations:** 1 Medizinische Klinik und Poliklinik I, Klinikum der Universität München, Ludwig-Maximilians-Universität, Marchioninistrasse 15, 81377 Munich, Germany; 2 DZHK (German Centre for Cardiovascular Research), Partner Site Munich Heart Alliance, 80802 Munich, Germany; 3 Department of Nuclear Medicine, Ludwig-Maximilians-University Munich, University Hospital, Marchioninistr, 15, 81377 Munich, Germany; 4 Ambulatory Healthcare Center Dr. Neumaier & Colleagues, Radiology, Nuclear Medicine, Radiation Therapy, Regensburg, Germany; 5 Die Radiologie, Munich, Germany

**Keywords:** Cardiac positron emission tomography, 18F-FDG, Myocardial infarction, Ischemia–reperfusion injury, Heart function

## Abstract

**Objective:**

Animal models for myocardial injuries represent important cornerstones in cardiovascular research to monitor the pathological processes and therapeutic approaches. We investigated the association of 18F-FDG derived left ventricular metabolic volume (LVMV), defect area and cardiac function in mice after permanent or transient ligation of the left anterior descending artery (LAD).

**Methods:**

Serial non-invasive ECG-gated 2-deoxy-2-[18F]fluoro-d-glucose positron emission tomography (18F-FDG PET) after permanent or transient LAD ligation enabled a longitudinal *in vivo* correlation of 18F-FDG derived left ventricular metabolic volume to functional parameters and myocardial defect.

**Results:**

The LVMV shows a more prominent drop after permanent than transient LAD ligation and recovers after 30 days. The loss of LVMV correlates with the defect area assessed by QPS software. Cardiac function parameters (e.g., EDV, ESV, SV) by the QGS software positively correlate with LVMV after permanent and transient LAD ligation.

**Conclusions:**

This study provides novel insight into 18F-FDG derived LVMV after permanent and transient LAD ligation by longitudinal in 18F-FDG PET imaging and underlines the associations of the FDG derived parameter and cardiac function.

**Supplementary Information:**

The online version contains supplementary material available at 10.1007/s12149-022-01734-8.

## Background

Coronary artery disease (CAD), the manifestation of pathological accumulation of atherosclerotic plaques in cardiac arteries, is a leading cause of morbidity and mortality in western society [1].

Narrowing the coronary arteries over time and rupturing atherosclerotic plaques result in myocardial injury by the diminished heart blood supply. Loss of cardiac muscle tissue leads to decreased cardiac function and can progress to heart failure with detrimental effects on the patient’s health. Percutaneous coronary intervention (PCI), a minimally invasive procedure to recanalize the occluded artery, facilitates reperfusion of the ischemic cardiac tissue and has significantly improved the outcome of patients after myocardial infarction [2].

To further reduce mortality and morbidity after ischemic cardiac injury, basic animal models are essential to understand the pathological processes better and transfer new therapeutics into the clinical setting. Nowadays, two surgical myocardial infarction models in mice are established to mimic cardiac injury [3, 4]. Permanent ligation of the LAD resembles a main cardiac artery's occlusion without timely reperfusion and represents a model for myocardial infarction (MI). The transient ligation of the LAD transfers the setting of early recanalization of an occluded coronary vessel into a suitable animal model (IR injury). While reperfusion improves the survival of ischemic cardiomyocytes, it also leads to a clinically relevant reperfusion injury (reviewed in [5]).

Besides cardiac magnetic resonance imaging, which is another valuable tool in mice as demonstrated by several investigators [6–8], the small animal PET imaging is an established modality in different disease models, e.g., ischemic [9–11] and dilated cardiomyopathy [12] and in pressure-overload left ventricular hypertrophy [13].

Regardless of a large amount of published data in the setting of ischemic cardiomyopathy, our study is the first to evaluate the association of LVMV and cardiac function after transient and permanent LAD ligation.

## Materials and methods

### Animals

Male C57/BL6 mice were purchased from Charles River (Sulzfeld, Germany). Animal care and all experimental procedures were performed according to the Guideline for the Care and Use of Laboratory Animals published by the U.S. National Institutes of Health (NIH publication no. 85–23, revised 1996). Myocardial infarction and ischemia–reperfusion injury were induced in 12–16 week-old C57BL/6 mice by surgical permanent (*n = *10) or transient (*n = *9) ligation of the left anterior descending artery, as described previously [3, 4]. The ECG of one mouse (MI group) was erroneous during the scan, and thereby no cardiac function parameters could be assessed. Healthy animals served as a control group (*n = *8). In brief, mice were anaesthetized using intraperitoneal injection of fentanyl (0.05 mg/kg), midazolam (5.0 mg/kg), and medetomidine (0.5 mg/kg), and mechanically ventilated (MiniVent Ventilator model nr. 845, Harvard Apparatus^®^). After left lateral thoracotomy, the proximal LAD was either ligated permanently (inducing MI) or transiently for 60 min, inducing IR injury. Study protocols complied with the institution’s guidelines and were approved by the Government’s animal ethics committee (ROB-55.2-2532.Vet_02-19-17 and ROB-55.2-2532.Vet_02-19-1).

### *In vivo* cardiac PET imaging

ECG-gated 18F-FDG-PET scans were performed on post-operation day 6 and day 30, using a dedicated small-animal PET scanner (Inveon Dedicated PET, Preclinical Solutions, Siemens Healthcare Molecular Imaging, Knoxville, TN, USA) as described previously [13, 14]. The animals had free access to food **(**standard laboratory chow from Ssniff Spezialdiäten GmbH, Soest, Deutschland) and water, as described previously [9, 12–15]. Anesthesia was induced (2.5%) and maintained (1.5–2%) with isoflurane delivered in pure oxygen at a rate of 1.5 L/min via a face mask. The core body temperature was maintained within the normal range using a heating pad and monitored by a rectal thermometer. Neonatal ECG electrodes (3 M, St. Paul, MN, USA) were placed on both forepaws and the left hind paw. Vital parameters were monitored and recorded using a dedicated physiological monitoring system (BioVet; Spin Systems Pty Ltd., Brisbane, Australia) [16]. After placing an intravenous catheter into a tail vein, approximately 20 MBq of 18F-FDG was injected in a volume of ~ 0.1 ml. The catheter was then flushed with 0.05 ml of saline solution. Animals remained anaesthetized during the entire scan and were placed in a prone position within the PET tomograph. A three-dimensional PET recording was obtained in list mode from 30 to 45 min after injection of the tracer. For attenuation and scatter correction, a 7-min transmission scan was performed with a rotating [57Co] source immediately after each PET scan, as described previously [12]. Recovery from anesthesia and the PET scan was monitored closely in the home cage by a veterinarian. The recorded data were processed with the Inveon Acquisition Workplace (Siemens Medical Solutions, Knoxville, TN, USA). FDG list-mode acquisitions were reconstructed, as described previously [12]. Reconstruction was performed using an OSEM 3D algorithm with 4 iterations (16 subsets) and a MAP 3D algorithm with 32 iterations (16 subsets) in a 128 × 128 matrix with a zoom of 211%. Data were either reconstructed as a static image or as gated images with 16 bins, normalized, corrected for randoms, dead time, and decay, as well as attenuation and scatter.

### PET image analysis

Analysis of PET images was performed by the Inveon Research Workplace (Siemens Medical Solutions) described previously [17, 18].

Inveon Research Workplace was used for assessing the percentage of the cardiac injected dose per gram (%ID/g) and left ventricular metabolic volume (LVMV) from static PET images. A cubic volume of interest (VOI) was drawn around the left ventricle, and a threshold value excluding 30% of the least hot voxels was applied. Correct VOI placement was verified in three projections (axial, sagittal, and coronal) [13]. In addition, ECG trigger signal accuracy was retrospectively verified using in-house software programmed in MATLAB (The Mathworks, Natick, USA) and C programming language [19].

The defect parameter was calculated with QPS^®^ (Cedars-Sinai, Los Angeles, CA, USA) using a normative database, as described previously [20]. In brief, infarct sizes were estimated with commercially available software (QPS^®^ 2012) from static FDG PET scans by creating polar maps of the left ventricle. In this approach, the FDG uptake in a given region is quantified as the percentage of the maximum tracer uptake in the polar map. The individual polar map is then compared to the average polar map from our normative database consisting of gender, strain, and age-matched animals (18 in total). The extent of the abnormally viable myocardium is calculated as a percentage of the left ventricle surface area.

Furthermore, the severity of the injured cardiac tissue is calculated in units of standard deviations below the normal tracer uptake. Extent and severity are combined into a single parameter corresponding to the infarct size expressed as a percentage of the left ventricular surface area. We used a similar approach as described by Slomka et al. [21] for myocardial perfusion data.

Left ventricular function parameters: end-diastolic (EDV), end-systolic (ESV) and the stroke volume (SV), and the left ventricular ejection fraction (EF), were calculated from ECG-gated images using QGS^®^ (Cedars-Sinai, Los Angeles, CA, USA), as described previously [12, 17].

### Histology

On day 30, mice were sacrificed, and the hearts were excised. After fixation in 4% phosphate-buffered formalin, hearts were cut into 2 mm thick slices and embedded in paraffin. Five micrometer thick sections were cut and mounted on positively charged glass slides. Standard histological procedure (Sirius Red/Fast green Staining) was performed as described previously [22].

### Statistical analysis

All results were expressed as means with standard deviation. One-way ANOVA analysis with Tukey’s multiple comparisons, unpaired Student’s *t *tests were used where appropriate. For groups without normal distribution, the Wilcoxon signed-rank or the Mann–Whitney *U* test was applied. The differences were considered statistically significant at a *P *value of 0.05.

## Results

### Defect size after transient and permanent LAD ligation using 18F-FDG PET imaging

The mouse hearts were evaluated using 18F-FDG PET imaging at days 6 and 30 post-transient or permanent LAD ligation. The defect assessment revealed a substantial defect in both models.

**Fig. 1:**
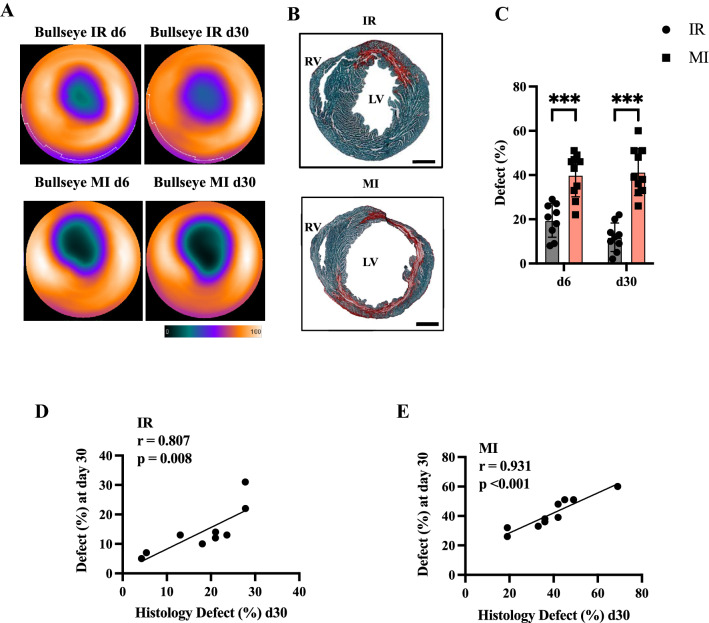
Assessment of myocardial defect in 18F-FDG PET imaging after transient and permanent LAD ligation. **A** Representative polar bulls-eye image of the left ventricle in IR day 6 and day 30 (upper images) and MI day 6 and day 30 (lower images) showing the defect by diminished 18F-FDG uptake. Colour Scale from QPS (Cool and in percentage range). **B** Representative histology section of hearts 30 days after IR (upper panel) and MI (lower panel) LAD ligation. Right ventricle (RV), left ventricle (LV). Sirius Red fast green staining of the left ventricle indicates the defect area. Bar equals 100 µm. **C** Quantification of the left ventricular defect after IR injury (in grey) and MI (in rose). **D** and **E** Correlation of histological defect to PET defect at day 30 after IR and MI injury. All groups: *n = *9–10. All data represent mean ± SD. *p =  0.05, **p <  0.01, ***p <  0.001

Permanent LAD ligation results in larger defect areas than transient ligation (illustrated in polar Bulls-eye map Fig. 1A, histology Fig. 1B and Fig. 1C, IR d6 vs MI d6, *p <  *0.001). Figure 1 B illustrates the histological evaluation after transient and permanent LAD ligation. The Sirius Red/Fast green staining was used to illustrate the fibrotic scar after injury. The transient LAD ligation leads to a smaller defect in the LV, due to the re-established perfusion of the infarct area. The defect after the permanent LAD ligation is far more severe and more cardiac mass is lost by the persistent obstruction of the LAD.

The defect among the two surgical approaches remained the same at 30 days post-operation (IR d30 vs MI d30, *p <  *0.001, Fig. 1C).

Neither after permanent nor after transient LAD ligation, a relevant change in the defect could be detected from day 6 to day 30 (MI d6 vs MI d30, ns; IR d6 vs IR d30, ns). Figure 1D and E is depicting the correlation of the histological defect assessment towards the PET defect in the IR model (*r =   *0.807, *p =  *0.008) and the MI model (*r =   *0.931, *p <  *0.001).

### Dynamic changes of LVMV and %ID/g after myocardial injury

Serial cardiac PET imaging enabled evaluating the left ventricular metabolic volume (LVMV), which is an 18F-FDG derived parameter [13] and the cardiac percentage of injected dose (%ID/g).

**Fig. 2:**
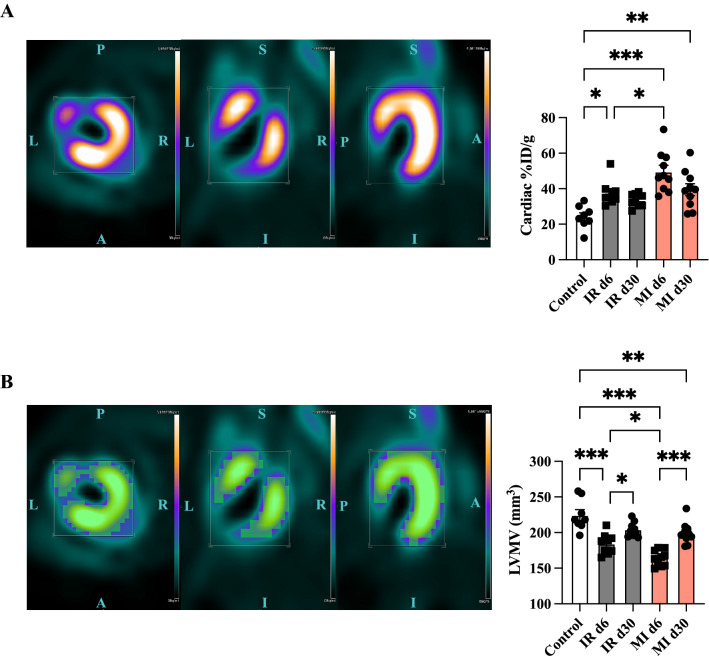
Alterations in LVMV and cardiac %ID/g after transient and permanent LAD ligation.** A** Representative static image after permanent LAD ligation exported from Siemens Inveon Workplace in different axes (left (L), right (R), planar (P), anterior (A), inferior (I), and sagittal (S). Bar equals uptake in from 0 to 5.9 × 10^6^ Bq/ml. Quantification of the cardiac %ID/g in the different groups on the right side. Control (in white), MI (in rose), and IR (in grey). All groups: *n = *8–10. All data represent mean ± SD. *p =  0.05, **p <  0.01, ***p <  0.001. **B** Illustration of the LVMV (in green) after permanent LAD ligation. Quantification of the LVMV after transient and permanent LAD ligation is shown on the right side. All groups: *n = *8–10. All data represent mean ± SD. *p =  0.05, **p <  0.01, ***p <  0.001

**Fig. 3:**
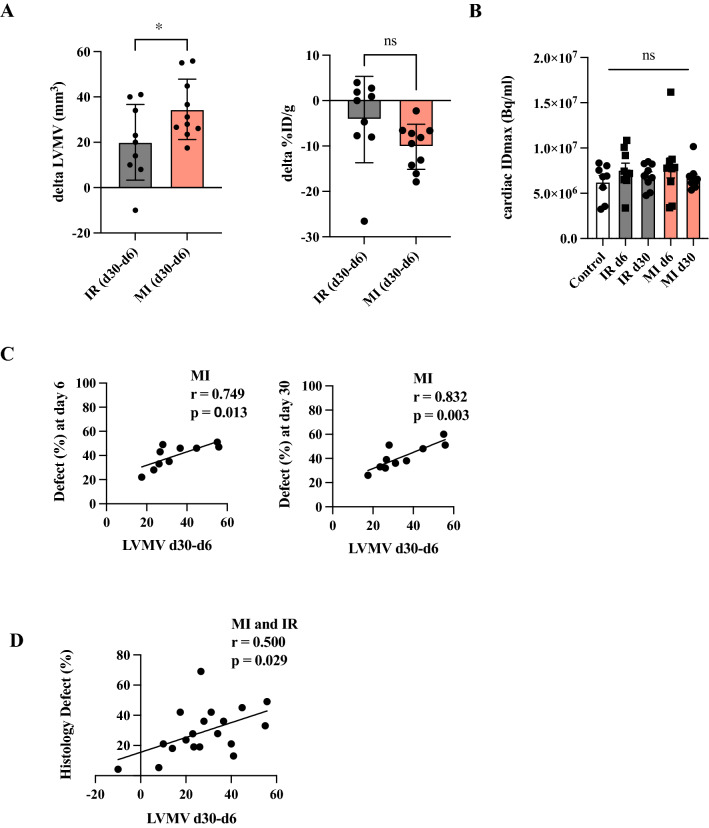
Increasing LVMV from day 6 to day 30 and correlation to defect area. **A** Difference in LVMV and %ID/g from day 30 to day 6 among the two cardiac injury models. **B** Quantification of the maximum ID/g among the different experimental groups. **C** Correlation of LVMV d30-d6 to defect area at day 6 (on the left side) and to day 30 (on the right side). All groups: *n = *8–10. All data represent mean ± SD. *p =  0.05, **p <  0.01, ***p <  0.001. **D** Correlation of LVMV d30-d6 to histological defect area after MI and IR injury

The cardiac %ID/g increased in both models compared to the control mice (control vs IR d6, *p* = 0.019; control vs MI d6, *p <  *0.001, Fig. 2A) and remained elevated at day 30 after permanent LAD ligation (control vs MI d30, *p* = 0.004). Regarding the %ID/g change, we could not detect any significant alterations from day 6 to day 30 (IR d30-d6 vs MI d30-d6, ns, Fig. 3A).

LVMV in both IR and MI models was decreased at day 6 compared to the control group and LVMV was reduced in IR compared to MI at day 6 (control vs IR d6, *p <  *0.001, control vs MI d6, *p <  *0.001, IR d6 vs MI d6, *p* = 0.042, Fig. 2B).

We observed increased LVMV in both models at day 30 (IR d6 vs IR d30, *p =  *0.046; MI d6 vs MI d30, *p <  *0.001). No difference in LVMV in the two injury models after 30 days was observed (IR d30 vs MI d30, ns).

We further calculated the change of LVMV from day 6 to day30 (Fig. 3A). Here we detected an increase in LVMV after permanent compared to transient LAD ligation (IR d30-d6 vs MI d30-d6, *p =  *0.049). Of note, we could not detect any difference in the maximum injected dose in the heart (Fig. 3B).

Comparing the defect and the change in LVMV displayed a significant correlation after permanent LAD ligation (day 6: *r =   *0.749, *p =  *0.013; and day 30: *r =   *0.832, *p =  *0.003; Fig. 3C). There was no correlation among the defect after transient LAD ligation and the change of LVMV from day 6 to day 30 (Data not shown). Figure 3D illustrates the correlation of the histological defect with the change in LVMV in both model (*r =   *0.500, *p =  *0.029).

### Left ventricular dilatation and diminished EF after permanent LAD ligation

Next, we assessed the alterations in cardiac volumes and function parameters to further enable the correlative analysis to the LVMV after surgical LAD ligation.

**Fig. 4:**
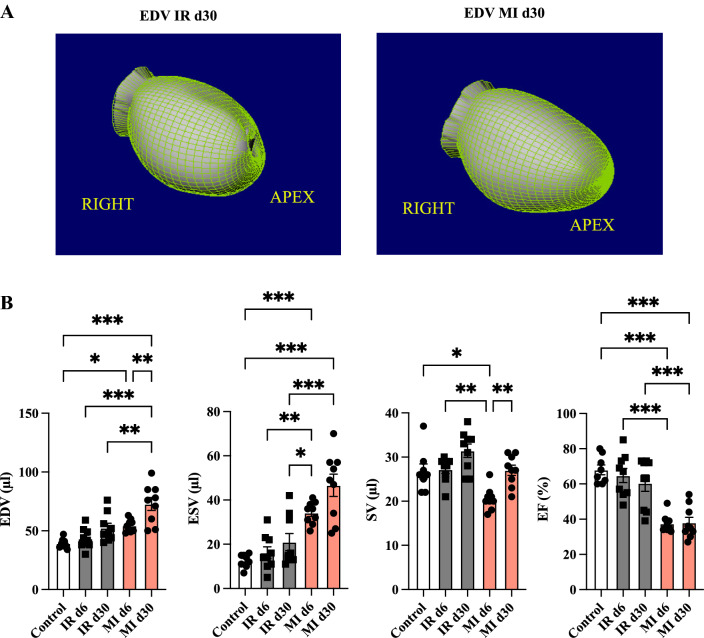
Longitudinal functional left ventricular parameters. **A** Illustrates three-dimensional evaluation from QGS software of the left ventricular EDV in the RAO view. Left picture: EDV at IR day 30 and right picture: EDV at MI day 30 illustrating the LV dilation. **B** Comparison of cardiac volume and function parameters at day 6 and d 30. Control (in white), IR (in grey) and MI (in rose). All groups: *n = *8–10. All data represent mean ± SD. *p =  0.05, **p <  0.01, ***p <  0.001

We detected an increased EDV on day 6 after permanent LAD ligation compared to the control and the transient ligation (Fig. 4A showing the 3D-reconstruction and quantification from QGS software, Fig. 4B, control vs MI d6, *p =  *0.037).

At day 30, the EDV further increased after MI, demonstrating ongoing LV dilatation (control vs MI d30, *p <  *0.001; MI d6 vs MI d30, *p =  *0.005; IR d30 vs MI d30, *p =  *0.002). The EDV in the reperfusion model increased but not achieve statistically significance over time (IR d6 vs IR d30, ns).

Additionally, we could note changes in the ESV (control vs MI d6, *p <  *0.001; control vs MI d30, *p <  *0.001; IR d6 vs MI d6, *p =  *0.003; IR d30 vs MI d30, *p <  *0.001; Fig. 4B).

The SV decreased after MI at day 6 (control vs MI d6, *p =  *0.012; Fig. 4B) and increased over time (MI d6 vs MI d30, *p =  *0.0061).

In line with the defect after MI, the EF was significantly lower after permanent LAD ligation than IR injury (control vs MI d6, *p <  *0.001; control vs MI d30, *p <  *0.001; IR d6 vs MI d6, *p <  *0.001; IR d30 vs MI d30, *p <  *0.001, Fig. 4B). After transient ligation, the EF was preserved after day 6 (control vs IR d6, ns; control vs IR d30, ns).

Furthermore, we could detect no improvement in the EF after 30 days of follow-up (IR d6 vs IR d30, ns; MI d6 vs MI d30, ns). Correlations of the cardiac function to the histological defect such as positive correlation to EDV (*r =   *0.618, *p =  *0.006), ESV (*r =   *0.626, *p =  *0.005), and negative correlation to EF (*r =   *-0.563, *p =  *0.015) underlining the methods itself are displayed in the supplement.

### Correlation of LVMV to cardiac function parameters

Finally, we evaluated the correlation of LVMV to cardiac volume and function estimated by the QGS software for both models.

**Fig. 5:**
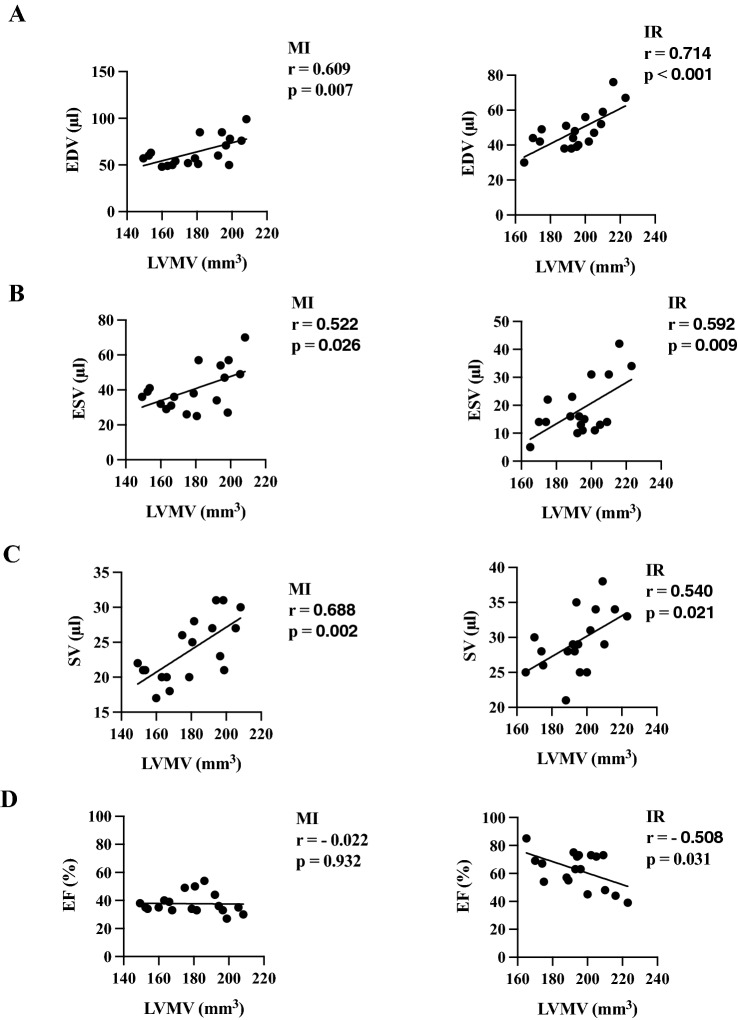
Correlation of LVMV to %ID/g and cardiac function parameters. Correlation of LVMV to EDV **(A)** after MI and IR injury. Data resemble cumulative day 6 and day 30. Illustration of the correlation of ESV **(B)**, SV **(C),** and EF **(D)** to LVMV after permanent and transient LAD ligation. Pearson correlation was used for all analyses

We could detect a positive correlation after permanent LAD ligation to EDV (*r =   *0.609, *p =  *0.007), ESV (*r =   *0.522, *p =  *0.026) and SV (*r =   *0.688, *p =  *0.002) (Fig. 5).

After transient LAD ligation, we as well detected several positive correlations: EDV (*r =   *0.714, *p <  *0.001), ESV (*r =   *0.592, *p =  *0.009), and SV (*r =   *0.540, *p =  *0.021)**.**

## Discussion

This study provides novel insight into 18F-FDG derived LVMV in two established models of ischemic cardiac injury using small-animal PET imaging [3, 4]. Using this experimental approach enables the multiparametric assessment of cardiac volume, function, and defect in a single modality, while otherwise the combination of PET and magnetic resonance imaging (MRI) or additional echocardiography is needed.

We aimed to evaluate the cardiac chamber volumes and function, the % ID/g and the LVMV, which previously was shown to correlate with fibrosis, end-diastolic volume and cardiac mass after pathological cardiac hypertrophy induced by transaortic constriction [13].

The permanent ligation of the LAD is a widely used model for myocardial infarction. However, it does not translate into the effect of myocardial reperfusion therapy by percutaneous coronary intervention which is widely available. Indeed, the cardiac IR model efficiently translates the revascularization after MI into mouse experiments and differences exist not only in functional data but also triggers a substantially different immune response compared to permanent LAD ligation [23].

First, we evaluated both models by assessing the defect area, showing an efficient ligation for both cardiac injury models. As expected, we detected positive correlation of the histological infarct area to EDV and ESV, and negative correlation to EF, indicating that larger histological defect result in LV dilatation and decrease in LV function.

Further, we found an initial drop in LVMV in both models, more pronounced after permanent LAD ligation, that in line with the more significant defects.

The defect achieved in our experiments matches the extent of previously published data [24]. The LVMW in both injury models increases over time, which could reflect cardiac remodelling after ischemic injury [13]. These observations were more pronounced after permanent LAD ligation, and the difference of LVMV from day 30 to day 6 correlated with the histological defect area. The defect or rather the fibrotic scar remains after myocardial injury. The LVMV in the mouse model, however increases during the cardiac remodelling as part of potential compensatory processes to overcome the damage, which is further underlined by the positive correlations towards the increasing EDV and SV. Therefore, the LVMV and defect size are focusing on different aspects after cardiac damage in mice.

This emphasizes the potential of LVMV as a monitoring parameter for changes in heart after ischemic injury.

Our measurements demonstrated a transient increase in %ID/g at day 6 after permanent and transient LAD ligation, which may to a certain extent be associated with inflammatory processes. In this particular setting, the %ID/g provides information of the cardiac uptake of FDG. This uptake is however not limited to cardiomyocytes or fibroblasts, but also to immune cells that are invading after ischemic injury. The further increased %ID/g after the permanent LAD ligation compared to the transient ligations underlines the increased damage and potentially a more pronounced immune invasion**.**

Myocardial infarction causes an inflammatory response, that triggers a recruitment of various immune cells [23]. At day 3 post-MI there is an increase in neutrophils, but macrophages are the dominant inflammatory cell population after the first week post-injury. Fate mapping and single cell transcriptomic at steady state and after injury aim to decipher this complex process. There are proposed differences temporal and spatial immune response after myocardial infarction (reviewed in [25–27]).

Increased 18F-FDG uptake and invading immune cells in human hearts at day 5 after myocardial infarction was proposed before [28]. Data from 49 humans with ST-elevation myocardial infarct and 18F-FDG PET imaging 5 days after PCI showed an inverse correlation of cardiac 18F-FDG uptake with functional outcome after 6 months [29]. However, it should be mentioned that the FDG protocols in humans and mice cannot be easily transferred from one species to another but may provide hints for future directions.

However, since isoflurane narcosis, in contrast to ketamine/xylazine narcosis as illustrated by, e.g., Vasudevan et al. [9], Thackery et al. [30], leads to an enhanced FDG uptake in the heart, our methodical approach prevents further conclusions due this technical limitation. Nevertheless, despite the isoflurane enhanced FDG uptake in all groups, we detect differences among the experimental groups, which are indicative for the contribution of invading immune cells.

Furthermore, comparing the two infarction models by volume and functional parameters displays that permanent LAD ligation led to ventricular dilatation and diminished left ventricular function [2]. The ejection fraction after transient ligation remained stable, which could be explained by a milder subendocardial defect resulting in preserved ejection fraction. In our experiments, the SV is not reduced at later stages in the infarct models compared to control, as expected in individuals with compensated heart failure [31].

The correlation of LVMV and cardiac volume parameters (EDV, ESV, SV) provides new insight into this parameter. Thus, increasing cardiac volume could be related to higher LVMVs. The correlation of EDV and ESV to LVMV indicated the utility of this parameter to monitor the volume remodelling after myocardial infarction by either permanent or transient LAD ligation. While there is a negative correlation of EF in the IR group, no correlation could be observed after permanent LAD ligation. The LV dilation and therefore the cardiac remodelling after permanent LAD ligation is by far more prominent, which might result in dilated hearts with a persistent lower EF compared to the transient LAD ligation with re-established perfusion and a decent dilations and preserved EF. Therefore, the LVMV should be carefully assessed as it might be affected by the extent of myocardial damage. It remains a possible limitation of this parameter, that volumes might be correlated, while the ejection fraction might be affected by other intracellular changes in cardiomyocytes or structural alterations (e.g.: myofibrils or sarcomere composition). Estimation of the LVMV from static images and correlation towards gated images could further represent a technical limitation. Calculating the LVMV in each frame after gated reconstruction without the technical averaging in static pictures could be a future directive.

If this parameter could provide the beneficial insight in rats and pigs or can also be translated into other tracers remains to be elucidated in future investigations.

Our study suffers from several limitations.

Both models bear the limitation of using healthy and young mice to compensate for the loss of viable tissue. Humans develop coronary artery disease while ageing and the erupting coronary plaques occur later in life.

Further, we did not evaluate the metabolic shifts in substrate difference after cardiac injury [30, 32], which could interfere with the cardiac FDG uptake under isoflurane narcosis [33]. However, we could not find a significant difference in the maximum uptake in FDG in the control compared to the infarction models. The cardiac metabolism could also be influenced by anabolic and catabolic hormones such as insulin, insulin growth factor, or glucagon, and blood glucose levels which were not evaluated in this study. Nevertheless, we followed a strict routine of mouse handling and narcosis in the process to avoid potential bias [14, 15, 33].

## Conclusions

This study provides new insight into the 18-FDG derived parameter LVMV and cardiac function in longitudinal PET imaging in two mouse models of myocardial infarction.

## Supplementary Information

Below is the link to the electronic supplementary material.

Supplementary file1 (DOCX 57 KB)

## Data Availability

The authors confirm that the data supporting the findings of this study are available within the article and/or its supplementary materials.

## Abbreviations

ID/g max: Maximum injected dose per gram
18F-FDG: 2-Deoxy-2-[18F]fluoro-d-glucose
IR: Ischemia reperfusion
LAD: Left anterior descending artery
LVMV: Left ventricular metabolic volume
MI: Myocardial infarction
PET: Positron emission tomography
ROI: Region of interest
VOI: Volume of interest
