# Supplementary material for: Comparison of transient and permanent LAD ligation in mice using 18F-FDG PET imaging
Source: Ann Nucl Med. 2022 Mar 30;36(6):533–43. doi: 10.1007/s12149-022-01734-8 (PMC9132804; doi:10.1007/s12149-022-01734-8)
Supplement: Supplementary file 1 — Supplementary file1 (DOCX 57 KB) [file 12149_2022_1734_MOESM1_ESM.docx]

Supplemental Figure S1: Correlation of histology defect to cardiac function parameters

Correlation of histology defect to EDV (A), SV (B), ESV (C), and EF (D) after MI and IR injury. Cumulative data show results at day 30 in both models. Pearson correlation was used for all analyses.
